# Cardiac myosin contraction and mechanotransduction in health and disease

**DOI:** 10.1016/j.jbc.2021.101297

**Published:** 2021-10-09

**Authors:** Samantha K. Barrick, Michael J. Greenberg

**Affiliations:** Department of Biochemistry and Molecular Biophysics, Washington University School of Medicine, St. Louis, Missouri, USA

**Keywords:** myosin, cardiac muscle, cardiomyopathy, cardiac development, mechanotransduction, contractile protein, heart, cMyBP-C, cardiac myosin-binding protein C, DCM, dilated cardiomyopathy, DRX, disordered relaxed state, ELC, essential light chain, HCM, hypertrophic cardiomyopathy, hiPSC-CM, human induced pluripotent stem cell–derived cardiomyocyte, HMM, heavy meromyosin, IHM, interacting heads motif, LMM, light meromyosin, MHC, myosin heavy chain, OM, omecamtiv mecarbil, RLC, regulatory light chain, S1, subfragment-1, S2, subfragment-2, SRX, super relaxed state

## Abstract

Cardiac myosin is the molecular motor that powers heart contraction by converting chemical energy from ATP hydrolysis into mechanical force. The power output of the heart is tightly regulated to meet the physiological needs of the body. Recent multiscale studies spanning from molecules to tissues have revealed complex regulatory mechanisms that fine-tune cardiac contraction, in which myosin not only generates power output but also plays an active role in its regulation. Thus, myosin is both shaped by and actively involved in shaping its mechanical environment. Moreover, these studies have shown that cardiac myosin-generated tension affects physiological processes beyond muscle contraction. Here, we review these novel regulatory mechanisms, as well as the roles that myosin-based force generation and mechanotransduction play in development and disease. We describe how key intra- and intermolecular interactions contribute to the regulation of myosin-based contractility and the role of mechanical forces in tuning myosin function. We also discuss the emergence of cardiac myosin as a drug target for diseases including heart failure, leading to the discovery of therapeutics that directly tune myosin contractility. Finally, we highlight some of the outstanding questions that must be addressed to better understand myosin’s functions and regulation, and we discuss prospects for translating these discoveries into precision medicine therapeutics targeting contractility and mechanotransduction.

Cardiac myosin is best known as the molecular motor that powers heart contraction. It is a member of the myosin II family (also known as conventional myosin), which includes two-headed, filament-forming muscle and nonmuscle myosins. Although this “conventional” myosin has been studied for several decades, recent studies have revealed novel functions and regulatory mechanisms that are anything but conventional. It has become clear that cardiac myosin not only powers heart contraction but also plays pivotal roles in muscle regulation, development, and mechanotransduction. Dysfunction of myosin in these processes can lead to a range of cardiac diseases with different presentations and prognoses.

Here, we review the complex functions and regulatory mechanisms of cardiac myosin revealed by recent multiscale studies. We highlight the roles played by myosin-based contractility and mechanotransduction in supporting cardiac development and their dysfunction in disease. Finally, we discuss cardiac myosin as an emerging target for novel therapeutics developed for heart diseases.

### Cardiac myosin structure

Cardiac myosin is a heterohexameric protein consisting of two myosin heavy chains (MHCs), two regulatory light chains (RLCs), and two essential light chains (ELCs) (Fig. 1*A*). Regions of myosin are frequently labeled by names derived from proteolytic fragmentation, where limited proteolysis with chymotrypsin yields heavy meromyosin (HMM) and light meromyosin (LMM) fragments. HMM can further be digested to yield subfragment-1 (S1), monomeric myosin heads containing either one or two of the light chains depending on the protease used, and subfragment-2 (S2). Each MHC S1 head contains a nucleotide-binding pocket and an actin-binding interface. The head domain is followed by the light chain-binding domain, a single alpha helix that amplifies small conformational changes in the nucleotide-binding site into a large displacement known as the working stroke or power stroke. The light chain-binding domain is mechanically reinforced by an ELC and an RLC, where the ELC is proximal to the head domain. The light chain-binding domain is followed by the S2 region, which helps to dimerize the two MHCs. The flexibility and length of the S2 region may impact the unloaded shortening velocity of muscle (that is, the speed at which the muscle length decreases in the absence of external force) by imposing a drag load on filaments moving at high velocities (1, 2, 3). As we will discuss later, S2 also plays an important role in myosin autoinhibition. The S2 region is followed by the LMM fragment (also known as the tail), which contains a large coiled-coil tail domain that interacts with tails of other myosin molecules to form the bipolar thick filament.

**Figure 1:**
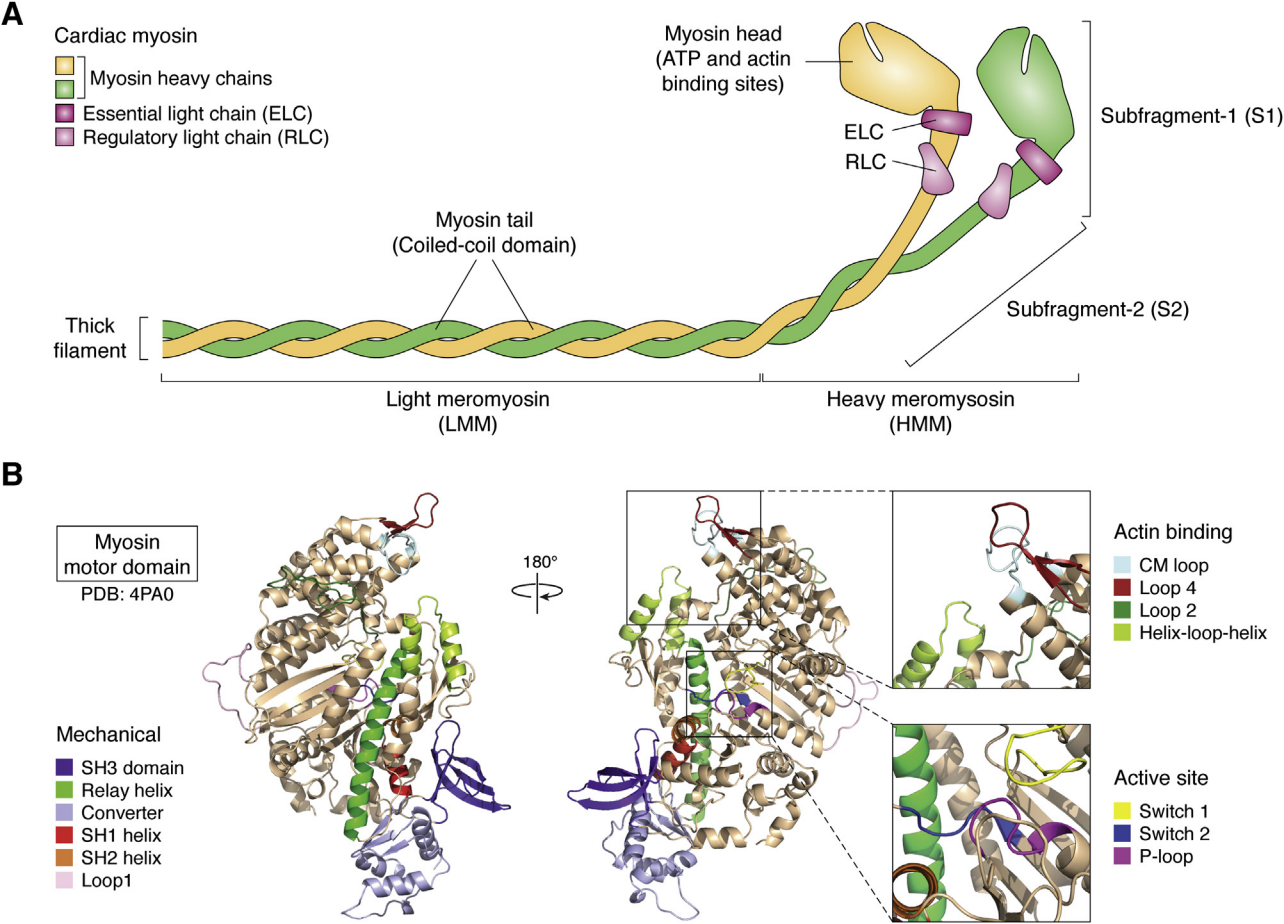
**Cardiac myosin structure.***A*, schematic structure of cardiac myosin showing the regions named for proteolytic fragments heavy meromyosin (HMM), light meromyosin (LMM), subfragment-1 (S1), and subfragment-2 (S2). Full-length myosin consists of two heavy chains (*yellow* and *green*), each bound to an essential light chain (ELC, *dark pink*) and a regulatory light chain (RLC, *light pink*). The myosin heads (S1 region) contain the sites of ATP and actin binding. The tails (LMM) are coiled coils that assemble into the thick filament. *B*, structure of the human cardiac myosin motor domain based on PDB ID 4PA0, where the loops not resolved in the crystal structure were modeled using homology modeling. Structural domains related to the mechanical power stroke, actin binding, and nucleotide binding to the active site are highlighted. For additional details on the structural mechanism of myosin force generation, the reader is referred to (7).

While many muscle and nonmuscle myosin isoforms have been crystallized (4, 5), it was not until 2015 that the first crystal structure of the cardiac myosin motor domain was solved (6). The myosin shares many of the critical structural elements common to myosins including loops involved in nucleotide binding (*e.g.*, P-loop, switch-1, and switch-2), actin binding (*e.g.*, the cardiomyopathy loop, loop 2, loop 4, and the helix-loop-helix domain), and mechanical elements (*e.g.*, relay helix, SH1 and SH2 helices, and converter domain) (Fig. 1*B*). The reader is referred to excellent reviews on myosin structure and function for additional details on the role of these elements in mechanochemical coupling (7, 8). Crystal structures of the cardiac myosin motor domain in both the pre-power stroke and post-power stroke conformations revealed a fold very similar to other myosin isoforms (6, 9, 10). This observation led to the question of how different myosin motors with high degrees of structural conservation can have biochemical properties (*e.g.*, ADP release rate, duty ratio) that vary by orders of magnitude. A recent molecular dynamics study demonstrated that small changes in myosin sequence can give rise to changes in global protein fluctuations, and that different conformational ensembles explored by a myosin motor in solution correlate with functionally relevant biochemical properties (11). Moreover, recent technological advances in the field of cryo-electron microscopy enabled the determination of a structure of cardiac myosin bound to actin (12). This structure revealed that several disordered loops in the myosin structure are stabilized in the actomyosin complex, including several residues frequently mutated in human disease.

### Cardiac muscle contraction

The fundamental contractile unit in cardiac muscle is the sarcomere, which consists of interdigitated thin and thick filaments that lie between α-actinin-containing structures called Z-discs (Fig. 2). Regions of the sarcomere are named for their appearance as light and dark bands in electron micrographs of muscle: myosin is localized to the dark A-band, whereas the light band that contains actin is called the I-band. Myosin-containing thick filaments are organized into A-bands by myomesin at the M-line and anchored to the Z-discs by titin. In the C-zone of the sarcomere, cardiac myosin interacts with cardiac myosin-binding protein C (cMyBP-C). cMyBP-C is anchored to the thick filament by its C-terminal region, whereas the N-terminal region regulates muscle contraction through its interactions with either the myosin head domain or the thin filament. Thin filaments, consisting of actin filaments decorated with proteins including tropomyosin and troponin, are anchored to the Z-discs *via* their barbed ends. This orientation of the actin filaments ensures that cardiac myosin, which moves toward the barbed end of actin, will directionally pull the Z-discs toward the center of the sarcomere, shortening the distance between the Z-discs.

**Figure 2:**
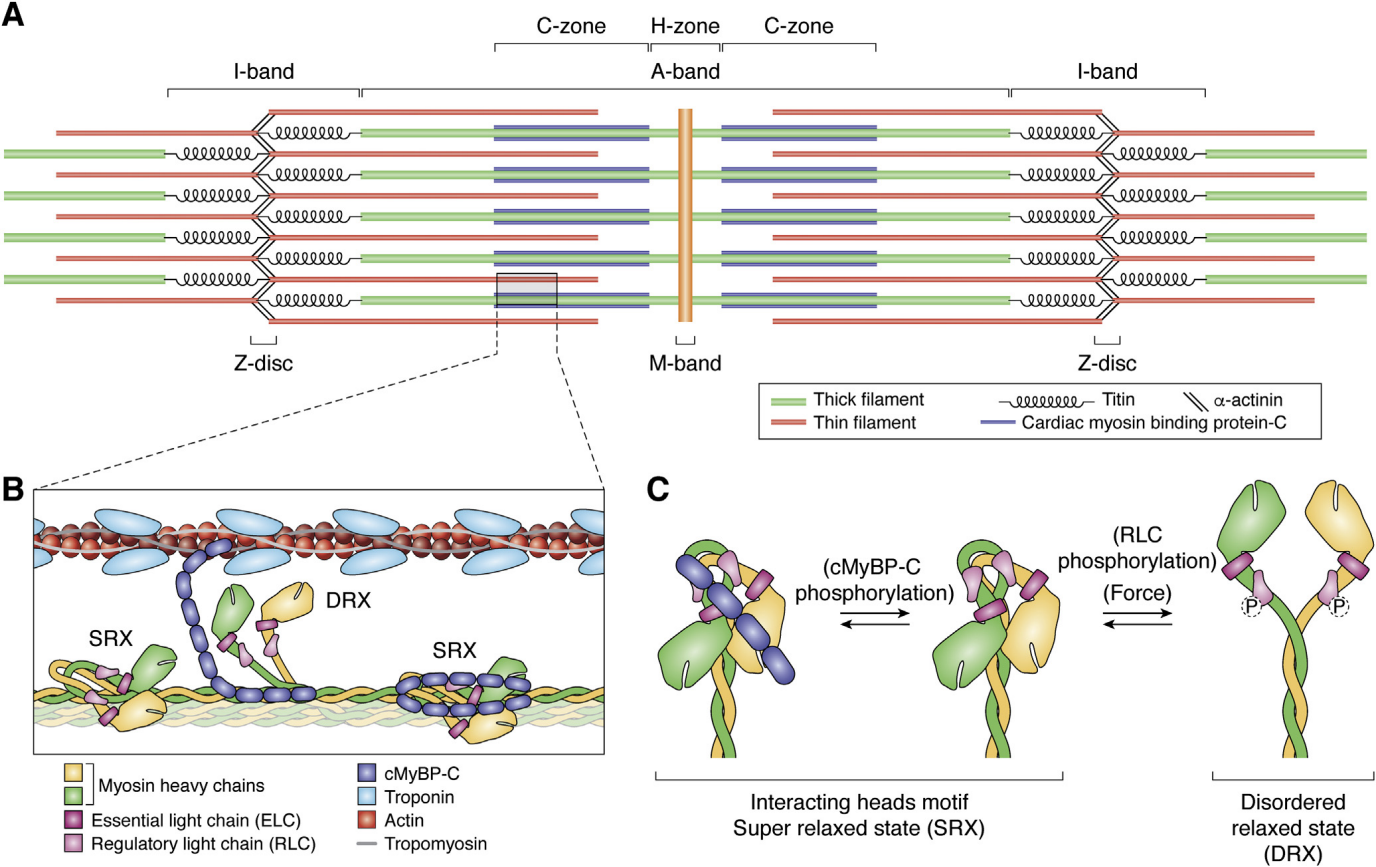
**Cardiac myosin integration into the sarcomere.***A*, diagram of the sarcomere, drawn roughly to scale. Bands and discs observed by electron microscopy are labeled. *B*, cartoon showing a zoomed-in view of the sarcomere within the C-zone. Elements are drawn roughly to scale. Myosin heads in the super relaxed state (SRX) are bound to the thick filament backbone, whereas myosin heads in the disordered relaxed state (DRX) are freed from the backbone. *C*, schematic structure of myosin in the autoinhibited, interacting heads motif (IHM) associated with the SRX (*left* and *center*) and the DRX (*right*). In the IHM, the blocked head (*yellow*) interacts with the free head (*green*). It has been proposed that the IHM is stabilized by cMyBP-C (*blue*), where phosphorylation of cMyBP-C reduces its interaction with myosin (*center*). Phosphorylation of the RLC (*pink*) and mechanical forces each promote the formation of the DRX; however, it is important to note that they are not required for the formation of the DRX. Note that RLC phosphorylation can occur on either one or both of the heads.

The force generated by a muscle is proportional to the number of actin-bound myosins (termed “crossbridges” because they bridge the thick and thin filaments) and the average force per crossbridge. Each individual motor generates 1 to 5 pN of force (13); however, the collective motion of many motors in the sarcomere produces higher forces. The number of crossbridges bound to actin at a given time is the product of the total number of available, active myosin heads, and the duty ratio (*i.e.*, the fraction of the biochemical cycle that a given crossbridge remains bound to actin) (14). The power output is the product of the shortening speed and the force. As such, force production depends on both the kinetics of the ATPase cycle and the mechanics of the motor. As discussed below, these parameters can be dynamically tuned to match the physiological loads of the body.

## The actomyosin biochemical cycle

**Figure 3:**
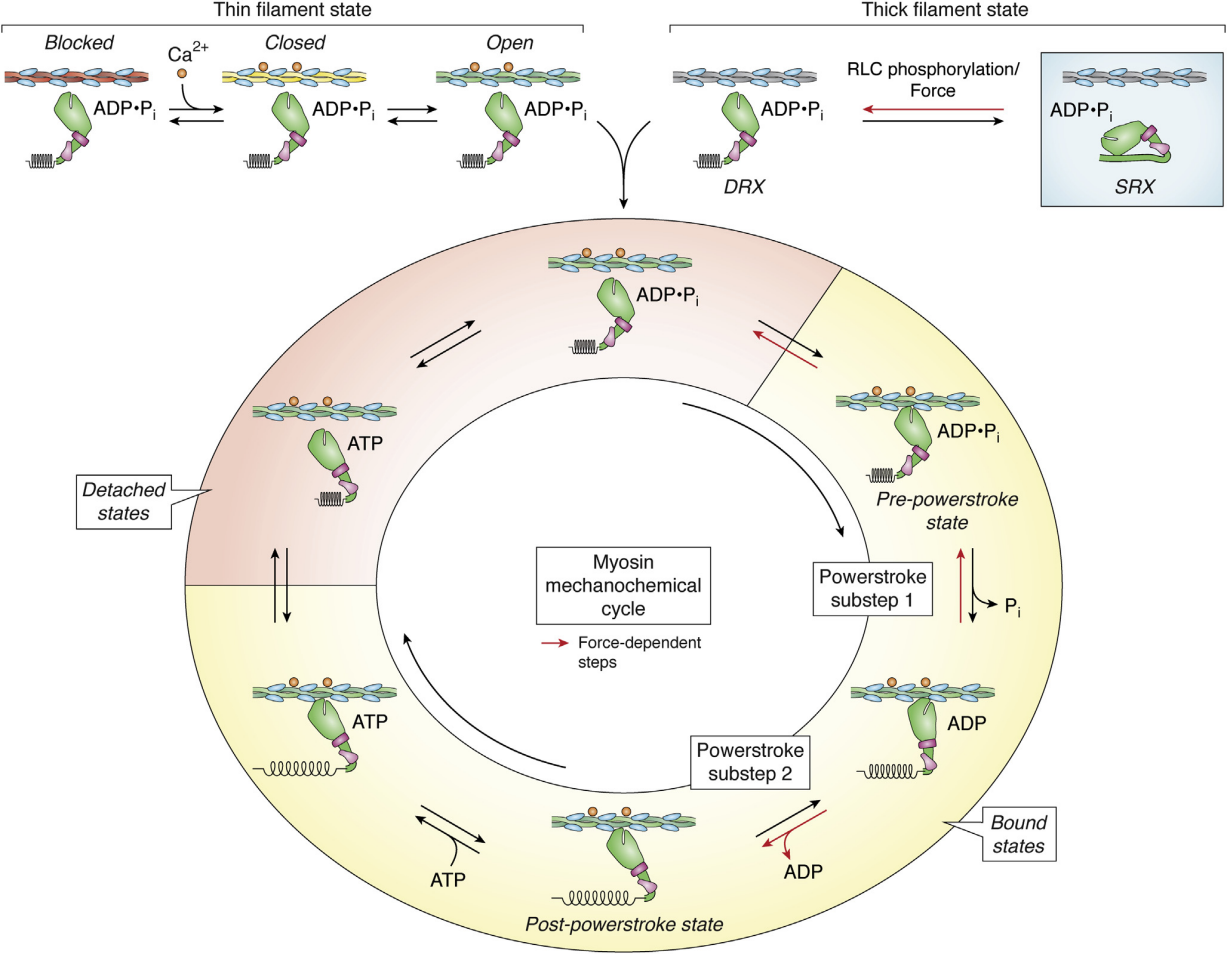
**The myosin mechanochemical cycle and its regulation.** For simplicity, only one myosin head is shown. In the super relaxed state (SRX, *blue shaded box*), myosin is autoinhibited. Force and RLC phosphorylation each promote the adoption of the disordered relaxed state (DRX). The thin filament can lie in the blocked, closed, or open states, where switching between states depends on calcium and myosin binding. In the open state, myosin can bind to and detach from the thin filament in a nucleotide-dependent manner. Bound and detached states are indicated by *yellow* and *red shading*, respectively. Force-dependent steps are indicated by *red arrows*. Mechanical substeps of the working stroke and structural states (pre-power stroke and post-power stroke) are labeled.

Myosin is an ATPase that utilizes energy drawn from ATP hydrolysis to generate movement (15) (Fig. 3). Changes in the myosin-bound nucleotide modulate the affinity of myosin for actin (16), where the myosin and actin can either be strongly bound (*i.e.*, force-generating or -bearing states) or weakly bound (*i.e.*, actomyosin is primarily dissociated but transiently interacting through short-lived, weak electrostatic interactions that do not bear load) (17, 18, 19). In the absence of nucleotide, myosin and actin are strongly bound in a rigor complex. ATP binding to actin-bound myosin causes actomyosin dissociation. Myosin hydrolyzes ATP to ADP and inorganic phosphate and isomerizes to the pre-power stroke conformation, where the head is primed to generate force. Myosin then rebinds to actin, initiating the force-generating power stroke and triggering the release of inorganic phosphate. During the power stroke, the light chain-binding domain rotates by ∼70°, generating a ∼4 nm displacement. ADP is then released and the light chain-binding domain rotates further to the post-power stroke state, generating an additional ∼2 nm displacement (20, 21) for a total displacement of ∼6 nm (22, 23, 24, 25, 26).

The transition to strong binding and the associated release of phosphate is the slowest step in the myosin ATPase cycle and the step associated with the largest release of free energy (16). Actin binding accelerates the rate of phosphate release from myosin by orders of magnitude; however, this transition remains the rate-limiting step of the overall ATPase cycle in the presence of actin (16). Although some structural studies have indicated that phosphate release precedes the power stroke (27, 28), recent optical trapping and spectroscopic measurements suggested that the power stroke precedes phosphate release (24, 29, 30, 31). Regardless of this order, the primary steady-state intermediate of the ATPase cycle is a state in which actin and myosin are dissociated and myosin is bound to ADP and inorganic phosphate.

While the rate of actin-activated phosphate release limits the overall ATPase cycle, other biochemical rates determine physiologically important parameters of muscle contraction, including the shortening speed, force, and power output. The unloaded shortening rate of a muscle is generally thought to be limited by the rate of actomyosin crossbridge detachment (32, 33). Therefore, the rate of ADP release sets the rate of unloaded shortening, since this rate limits crossbridge detachment at physiologically relevant ATP concentrations (34). The speed is also proportional to the size of the myosin working stroke (32), which can be altered by disease-causing mutations (21). It should be noted that other models of muscle contraction have been proposed, including a recent model where the speed depends on both attachment and detachment kinetics (2). In this model, the speed is related to the length and flexibility of the S2 linker as well as the myosin step size.

## Regulation of actomyosin-based force generation

While the basics of the myosin biochemical cycle have been understood since the 1970s (16), our understanding of the regulation of cardiac myosin has advanced greatly in the past few years. These advances have been made possible by multiscale studies of increasing complexity and several technological innovations. These studies have given a more nuanced understanding of how the heart fine-tunes its power output over various length and temporal scales, identified new mechanisms of disease pathogenesis, and provided new avenues for the development of therapeutics for heart diseases. Moreover, they identify mechanisms by which cardiac myosin not only generates cardiac power output, but also plays a critical role in its regulation.

### Regulation of accessibility of myosin-binding sites on actin

Cardiac contraction is regulated from beat-to-beat by calcium (Ca^2+^). The calcium-dependent interactions between the thick and thin filaments are regulated by troponin and tropomyosin (Figs. 2 and 3) (35, 36). During diastole, the relaxation phase of the cardiac cycle, intracellular calcium levels are low and tropomyosin adopts the blocked state, sterically blocking myosin-binding sites along the thin filament and preventing contraction. During myocyte activation, intracellular cytoplasmic calcium levels rise, and calcium binding to troponin C causes tropomyosin to move to the closed position on the thin filament, partially exposing the myosin-binding interface on actin. Tropomyosin can then move into the open position, in which the myosin-binding site is fully exposed. The binding of one myosin uncovers adjacent myosin-binding sites on actin that were obscured by tropomyosin, resulting in cooperative activation of the thin filament. Myosin generates force during systole, the contraction phase of the cardiac cycle, until cytoplasmic calcium levels drop, leading to deactivation of the thin filament. Thus, the processes of muscle activation and relaxation depend on both calcium and myosin binding to the thin filament (35, 37, 38, 39).

Recent studies have shown that myosin-binding protein-C (cMyBP-C) may play a role in tuning thin filament activation. The reader is referred to a recent review on cMyBP-C for more details on its structure and function (40). The N-terminal region of cMyBP-C can bind to the thin filament (41), shifting tropomyosin into the open position (42, 43, 44). This shift reduces the energy barrier for myosin strong binding, enabling force generation at lower levels of calcium (45). This is consistent with muscle fiber studies using cMyBP-C knockout mice (46) and mice lacking the N-terminal region of cMyBP-C (47), which each showed reduced thin filament activation in response to calcium. Moreover, elegant experiments using transgenic mice expressing an engineered construct of cMyBP-C with an N-terminal region that can be cleaved and exchanged with a recombinant cMyBP-C construct showed that removal of the N-terminal region causes spontaneous oscillatory contractions (47). These spontaneous oscillations were damped when the N-terminal region was added back to the cMyBP-C. Based on these results, it was proposed that cMyBP-C binding to the thin filament helps to maintain tropomyosin in the open position, preventing relaxation of the muscle before calcium levels drop (48). This activation can be dynamically tuned. Upon β-adrenergic stimulation, protein kinase A (PKA) phosphorylates cMyBP-C, reducing the binding of cMyBP-C to the thin filament and tuning the level of thin filament activation (49, 50). We speculate that myosin-induced movement of tropomyosin along the thin filament may also increase the binding of cMyBP-C to the thin filament, since it exposes cMyBP-C-binding sites along actin.

### Regulation of the number of force-generating myosin heads

Although the regulation of molecular motors by autoinhibition has been recognized for a while (51, 52, 53, 54, 55, 56), even in the context of myosin II (57, 58, 59, 60, 61), recognition of the functional importance of autoinhibition in striated muscle myosin has emerged more recently. Key evidence of thick filament-based regulation of striated muscle was provided by measurements in Roger Cooke’s lab of the single turnover rate of ATP in relaxed skeletal muscle (*i.e.*, at low calcium) using a fluorescent ATP analog, Mant-ATP (62). They observed biphasic turnover kinetics, with one rate similar to that of ATP turnover of myosin S1 in solution in the absence of actin (∼0.05 s^−1^) and a much slower rate (0.004 s^−1^). They proposed that the fast and slow rates represent populations of myosins in a disordered relaxed state (DRX) and super relaxed state (SRX), respectively (62, 63). The SRX was later observed in cardiac muscle (64).

It has been proposed that the biochemical SRX corresponds with an autoinhibited structural state observed in cryo-electron microscopy reconstructions of thick filaments from cardiac muscle, smooth muscle, and tarantula skeletal muscle (59, 65, 66, 67). In this structure, the myosin heads fold back toward the S2 region to form an asymmetric structure known as the interacting heads motif (IHM) (Fig. 2). One head, dubbed the blocked head, is locked in the pre-power stroke state, and it interacts with its own S2 region. The primary head–head interaction site, which is adjacent to the actin-binding region of the blocked head, interacts with the converter domain of the other, free head, locking both heads in the autoinhibited state (68, 69). Interestingly, this structure has many similarities to the autoinhibited state seen in isolated, full-length smooth muscle and nonmuscle myosin II molecules (58, 60, 61).

Several pieces of evidence connect the biochemical SRX and the IHM structural state. X-ray diffraction studies of intact cardiac muscle fibers demonstrated a correspondence between the fraction of ordered myosin heads along the thick filament backbone and the fraction of heads in the SRX measured biochemically (68). Negative stain electron microscopy of myosin motors also revealed a correlation between the fraction of heads in the SRX and the fraction of heads that form an autoinhibited structure. In addition, small-molecule compounds that altered the biochemical SRX induced a similar change in the fraction of heads in the autoinhibited state. Collectively, these data suggest a correspondence between the SRX and the IHM.

The IHM is likely stabilized by interactions between myosin and cMyBP-C (70). Spudich noted that the cardiac myosin head has a large, flat region enriched with charged residues that is likely involved in forming protein–protein interactions, which he dubbed the “mesa domain” (69). Many mutations that cause genetic cardiomyopathies are found on the mesa domain (71). Part of the mesa forms the interface of the IHM; however, another part of this region is exposed. It was proposed that the exposed part of the mesa interacts with cMyBP-C in the thick filament. Deletion of cMyBP-C reduces the formation of the SRX (72). Moreover, it was shown that an N-terminal fragment of cMyBP-C can bind to myosin in solution, and that this binding promotes the formation of the biochemical SRX (70). Phosphorylation of cMyBP-C by PKA upon β-adrenergic stimulation decreases the interactions between cMyBP-C and myosin, destabilizing the SRX (73).

The fraction of myosins in the SRX can be dynamically modulated by mechanisms that decrease the fraction of myosin heads in the SRX, increasing the number of myosin heads available to generate force (Figs. 2 and 3). For example, RLC phosphorylation by myosin light chain kinase is essential for the activation of smooth muscle and nonmuscle myosin II isoforms, but not striated muscle myosins. In the heart, cardiac contraction is modulated through a large gradient of RLC phosphorylation between layers of the heart that has been proposed to play a role in generating torque during blood ejection (74). X-ray diffraction studies demonstrated that RLC phosphorylation causes the myosin heads to move closer to the thin filament, and that this modulates the kinetics of muscle contraction (75, 76, 77). Recent studies demonstrated that RLC phosphorylation destabilizes the SRX, increasing the population of the DRX state (70).

The fraction of myosin heads in the SRX can also be regulated by mechanical force (78). Recent studies using intact muscle fibers demonstrated that stretching cardiac muscle causes an increase in the fraction of available heads (79, 80). This mechanosensitive release of myosin motors in the SRX from the thick filament backbone might enable the muscle to quickly and dynamically regulate the fraction of heads in the SRX during times of increased workload (80, 81). The force-dependent decrease in the SRX population results in generation of larger forces by myofilaments at longer sarcomere lengths, which, together with incompletely understood thin filament-based mechanisms, underlies length-dependent activation in cardiomyocytes (79, 80, 81, 82, 83, 84). Length-dependent activation contributes to the tissue-level relationship between the extent of ventricular filling and the strength of cardiac contraction, known as the Frank–Starling law of the heart (85). The force-induced destabilization of the SRX can only be observed in the context of myosin filaments, emphasizing the importance of multiscale studies to fully understand the mechanisms intrinsic to myosin that help to regulate and fine-tune the force and power output of muscle.

### Regulation of myosin motor function by mechanical forces

Mechanical forces that assist or resist the myosin power stroke can affect the rates of biochemical transitions, thereby tuning muscle force production and power output. This mechanical regulation tunes the myosin contractility on very fast timescales (*i.e.*, from power stroke to power stroke). This was first recognized in pioneering work by Hill and Fenn, who noted that the heat released during muscle shortening was greater during unloaded shortening than under high tension (86, 87). In the case of muscle myosins, muscle fiber and optical trapping experiments have revealed that there are multiple force-sensitive transitions (Fig. 3) (20, 24, 88, 89). As described earlier, the unloaded shortening speed of muscle is limited by the rate of ADP release, the transition that limits actomyosin dissociation at physiological ATP concentrations (20, 34, 90). In the presence of loads that oppose the myosin working stroke, the rate of ADP release slows, slowing the rate of muscle contraction (20, 25, 91).

In addition to slowing ADP release, mechanical forces can also modulate the number of force-generating crossbridges by affecting both the isomerization to the strong binding state and subsequent phosphate release (24, 88). Recent experiments using an ultrafast optical trap demonstrated that increasing loads that either resist or assist the power stroke can increase the rate of actomyosin dissociation before myosin isomerization to the strong binding state (Fig. 3) (24). This pre-power stroke detachment occurs at rates that are an order of magnitude faster than the rate of ADP release. Assisting and resisting forces have asymmetric effects on this dissociation rate due to the stereospecific interactions required for myosin cleft closure. Moreover, forces that resist the power stroke can also increase the probability of phosphate rebinding and reversal of the power stroke. Taken together, these three force-dependent pathways (slowing of ADP release, preventing strong binding, and reversing the power stroke) tune the speed, force, and power generated by the muscle.

### Regulation by transcriptional regulation of isoform expression

The contraction of cardiac myosin can be regulated transcriptionally by differential expression of MHC, ELC, and RLC subunits. In the adult human heart, the primary ventricular isoforms are *MYH7* MHC (β-cardiac myosin), *MYL3* ELC, and *MYL2* RLC, whereas the primary atrial isoforms are *MYH6* MHC (α-cardiac myosin), *MYL4* ELC, and *MYL7* RLC (92). While these are the primary isoforms expressed, multiple isoforms are expressed within a ventricle, and heteromolecules (*e.g.*, a myosin dimer made of one *MYH7* MHC and one *MYH6* MHC) have been observed (93). Evidence from murine hearts showed that the expression of *MYH6* and *MYH7* MHCs in the ventricles can vary spatially with enrichment in substructures within the heart (94). The exact composition of MHC, RLC, and ELC can tune the kinetics and mechanics of the myosin. For example, *MYH7* MHC has a significantly slower ADP release rate and unloaded shortening speed compared with *MYH6* MHC (95, 96), but *MYH7* motors can generate a larger ensemble force (97). Similarly, the size of the working stroke of muscle myosins depends on the ELC isoform (98).

The expression of myosin isoforms changes over time during development and disease. For example, in the human heart, the fraction of *MYH7* MHC expressed in both the atria and ventricles increases between the fetal and adult hearts (99). The fraction of *MYH7* MHC also changes during disease. In the ventricle, *MYH7* MHC expression increases from ∼93% in the healthy heart to ∼100% *MYH7* MHC in the failing heart (100, 101), and the amount of *MYH7* MHC in the atria increases from ∼14% in the healthy heart to ∼50% in the failing heart. Therefore, changes in the expression of different myosin isoforms play an important role in modulating cardiac contractility during long-term cardiac remodeling associated with development and disease.

## Myosin mechanosensing

Beyond its role generating cellular tension, cardiac myosin is part of the machinery necessary for sensing and transducing molecular forces. Thus, disrupted cellular tension affects cellular processes beyond muscle contraction. For example, myosin-generated tension is required to support sarcomere organization in cardiomyocytes, although the respective roles of cardiac and nonmuscle myosins in sarcomere assembly and maintenance remain somewhat controversial (102, 103). Treatment with blebbistatin, which disrupts myosin-driven contraction, leads to loss of sarcomere content in human induced pluripotent stem cell–derived cardiomyocytes (hiPSC-CMs) (102). Milder disruptions to myosin-generated force by pathogenic mutations in sarcomeric proteins can lead to sarcomeric remodeling (104, 105).

Myosin mechanosensing also plays a direct role in development. The stiffness of cardiac tissue increases during embryonic development, where adult tissue is ∼10× stiffer than the embryonic heart (106). The myosin-driven spontaneous contraction of cardiomyocytes isolated from embryonic chick hearts is optimized on substrate stiffnesses similar to that of embryonic heart tissues (107). Alteration of tissue elasticity *via* treatment of isolated embryonic heart tubes with collagenase (to soften) or a cross-linking enzyme (to stiffen) each impaired spontaneous beating, demonstrating that myosin contractile function is exquisitely matched to matrix elasticity (107). Moreover, myosin contractility plays a role in feedback loops of signaling pathways driving the cell cycle in embryonic hearts (108). Therefore, perturbations that affect myosin function can effect changes beyond force generation.

## Altered myosin contractility leads to disease

Given its central role in driving circulation, functional cardiac myosin is essential for life. Actomyosin function can be modulated by many factors, including posttranslational modifications (109, 110, 111, 112) and genetic mutations. A large number of mutations in cardiac myosin genes, including both the heavy and light chains, are associated with human disease, including familial cardiomyopathies (hypertrophic, dilated, and restrictive) (113, 114, 115), left ventricular noncompaction (116), skeletal myopathies (117), developmental defects (*e.g.*, atrial septal defects) (118), and electrophysiological disorders (*e.g.*, sick sinus syndrome) (119). The majority of these pathogenic mutations are point mutations that change myosin’s properties without rendering it nonfunctional, since major changes are incompatible with life. Interestingly, different mutations within the same gene can lead to different diseases, and the complexity of the resulting disorders clearly demonstrates that these mutations affect processes beyond just force generation. The variety of diseases caused by myosin mutations suggests that the context and specific effects of the mutation on molecular function are important for both the disease pathogenesis and progression (120). Below, we discuss two different classes of diseases caused by mutations in cardiac myosin.

### Familial cardiomyopathies

Familial cardiomyopathies, leading causes of sudden cardiac death and heart failure in young people, are caused by mutations in several genes, including those encoding myosin heavy chain (*MYH7*), ELC, and RLC. *MYH7* mutations were the first identified genetic causes of familial hypertrophic cardiomyopathy (HCM) (121, 122), a heart disease characterized by left ventricular hypertrophy and impaired cardiac relaxation (115). Since then, it has been shown that mutations in cardiac myosin (MHC, RLC, and ELC) (123) can also cause dilated cardiomyopathy (DCM), which involves left ventricular dilation and impaired systolic function (124).

A central challenge for the field is to distinguish primary drivers of disease from downstream and/or secondary effects (120). This is particularly challenging since these disorders are protean in nature, where different pathways are activated over the course of these progressive diseases (often years to decades). A mechanistic understanding is important for the design of potential precision medicine therapeutics. The involvement of mutations in myosin and other sarcomeric proteins (125, 126) in HCM and DCM suggests altered cardiac contractility as a pathogenic mechanism. The integral of tension development over time in both mice and hiPSC-CMs predicted whether a given mutation leads to hypertrophic or dilated cardiomyopathy better than measurements of calcium handling (127), where increased contractility is associated with HCM and decreased contractility with DCM. Importantly, modulation of the tension integral can prevent pathogenic remodeling (127, 128). Moreover, recent studies from our lab used a computational model of cardiac contraction (129) to show that altered thin filament regulation can predict changes in cardiac contractility that are consistent with those seen in cardiomyocytes (105).

Due to the inherent complexity of the disease pathogenesis, there has been a strong push to develop model systems that recapitulate the disease phenotype and enable functional studies of human disease-causing mutations *in vitro*. Excellent transgenic mouse studies have provided insights into the disease pathogenesis; however, they have the limitation that mouse ventricles express primarily *MYH6* MHC, not *MYH7* MHC like in the human heart (130). Due to functional differences between *MYH6* and *MYH7* MHCs, the presentation and biophysical defects associated with a given mutation can vary depending on the MHC backbone (131, 132, 133, 134, 135, 136). Although *MYH7* MHC protein can be obtained from patient biopsy samples, there are several associated challenges, including small quantities and unknown ratios of mutant to wild-type protein, which can lead to inconsistent results (137, 138). Thus, one of the principal challenges to studying human *MYH7* mutations *in vitro* has been the lack of recombinant expression system. While many myosin isoforms, including nonmuscle myosin-II and smooth muscle myosin, can be expressed using the baculovirus/Sf9 system, striated muscle myosins cannot due to the lack of muscle-specific chaperones (139, 140, 141). The recombinant expression of striated muscle myosins in murine myoblast cells (C2C12) was a major step forward that enabled the production of sufficient quantities of soluble recombinant protein for functional and structural studies (6, 95, 142).

Studies using recombinant human cardiac myosin revealed that cardiomyopathy-associated mutations can directly affect contractility at the level of individual motors (143, 144). Mutations that cause HCM typically increase molecular contractility, whereas DCM-causing mutations decrease contractility (123). However, some of these effects only become apparent when looking at reconstituted systems of increasing complexity. For example, studies using recombinant R403Q cardiac myosin in the *MYH7* MHC backbone observed increased velocity in an unregulated *in vitro* motility assay; however, the difference in velocity disappeared when regulated thin filaments were used (145). Furthermore, the intrinsic force generated by an R403Q myosin motor was lower than that generated by wild-type myosin, and altogether the results were consistent with reduced contractile function for R403Q motors (145). This might have suggested that hypercontractility in HCM caused by R403Q can be a secondary compensatory mechanism to counteract reduced myosin function, rather than a primary driver of disease. However, it was later demonstrated that the R403Q mutation destabilizes the myosin SRX, leading to increased contractility (70, 146). Furthermore, the R403Q mutation ablates binding of cMyBP-C, which promotes adoption of the SRX in wild-type myosin (146). This mechanism of increased contractility *via* an increase in the number of available myosin heads, regardless of the intrinsic contractility of each myosin head, has also been demonstrated for several other HCM-associated mutations in cardiac myosin (146, 147, 148, 149). In contrast, DCM-causing mutations tend to directly affect the force-generating capacity of individual myosin motors (150, 151). That said, the effects of a given mutation on protein function are often complex and may be best described by a combination of effects rather than a single mechanism. However, the grouping of mutations by common effects is useful for our fundamental understanding of disease pathogenesis as well as to aid the development of novel therapeutics that target specific disease mechanisms, as described in a recent review (120) and discussed below.

### Atrial septal defect

Mutations in *MYH6* MHC, the primary atrial myosin isoform, can lead to atrial septal defect, a congenital heart malformation (118, 152, 153). During development, *MYH6* is required for the proper formation of the atrial septum, the wall between the two atrial chambers (118). Mutations in *MYH6* that cause atrial septal defect appear to affect the interaction between the myosin heavy chain and the RLC (118) or between myosin and actin (152, 153). In contrast to cardiomyopathies, which can take decades to develop into symptomatic disease despite the presence of the mutation since birth, atrial septal defects are congenital heart defects. This is consistent with actomyosin-generated tension during development driving cellular function and organization.

## Altered contractility results in downstream mechanobiological dysfunction

Although pathogenic point mutations in cardiac myosin can affect cardiac contraction, changes in contractility in isolation are insufficient to explain the disease pathogenesis and complexity of presentation. At the molecular scale, defects in function due to pathogenic mutations are usually subtle, which is not surprising because major changes would be incompatible with life. In the case of familial cardiomyopathies, the mutant protein is expressed over the entire lifetime of the patient; however, pathological cardiac remodeling often does not occur until adolescence or later (154). There is evidence that genotype-positive patients show alterations in cardiac contractility before the development of pathogenic remodeling and symptomatic disease (155, 156). As such, mutation-induced changes in myosin contractility can be the initial insults that drive the early disease pathogenesis, leading to the activation of adaptive pathways that initially normalize cardiac function, but eventually transition to maladaptive remodeling. These maladaptive responses drive the disease symptoms and progression. Altered calcium handling (157), increased fibrosis (158), myofibrillar disarray (159), aberrant electrophysiology (160), and cellular hypertrophy (161) are all downstream consequences of the initial insult of altered contractility.

Similarly, in the case of atrial-septal defects caused by *MYH6* mutations, changes in myosin-based force production affect cardiac development. In this case, the disease phenotype is likely due to changes in the interactions between cells, not to changes in ejection fraction. The alteration of cellular function, gene expression, and tissue morphology by mutation-induced changes in myosin-based tension generation highlight the important role that cardiac myosin plays in mechanobiological signaling pathways.

Pathogenic mutations in myosin can also drive changes in tissue mechanics. Altered tissue stiffness plays a role in heart failure, where the prevalence of detyrosinated microtubules results in increased cardiomyocyte stiffness and impaired contractility (162). Higher levels of microtubule detyrosination were found in tissue isolated from HCM patients with sarcomeric mutations compared with sarcomere mutation-negative HCM patients, suggesting that microtubule detyrosination plays a role in the downstream pathogenesis of HCM caused by mutations in sarcomeric proteins (163).

Finally, it is important to note that disrupted force generation can affect processes beyond the myocyte. An example of this is the activation of the fibroblast-to-myofibroblast transition by mechanical tension (164). Multiple stimuli that alter cardiac contraction (*e.g.*, hypertension, mutations, aortic stenosis) can drive the development of hypertrophy and myofibroblast activation. Myofibroblasts secrete large amounts of extracellular matrix, contributing to fibrosis frequently seen with cardiomyopathies (165). Therefore, when considering the downstream effects of myosin mutations on disease, it is important to consider that these effects can be long-lasting and extend beyond the myocyte.

## Small molecules that target myosin contractility

**Figure 4:**
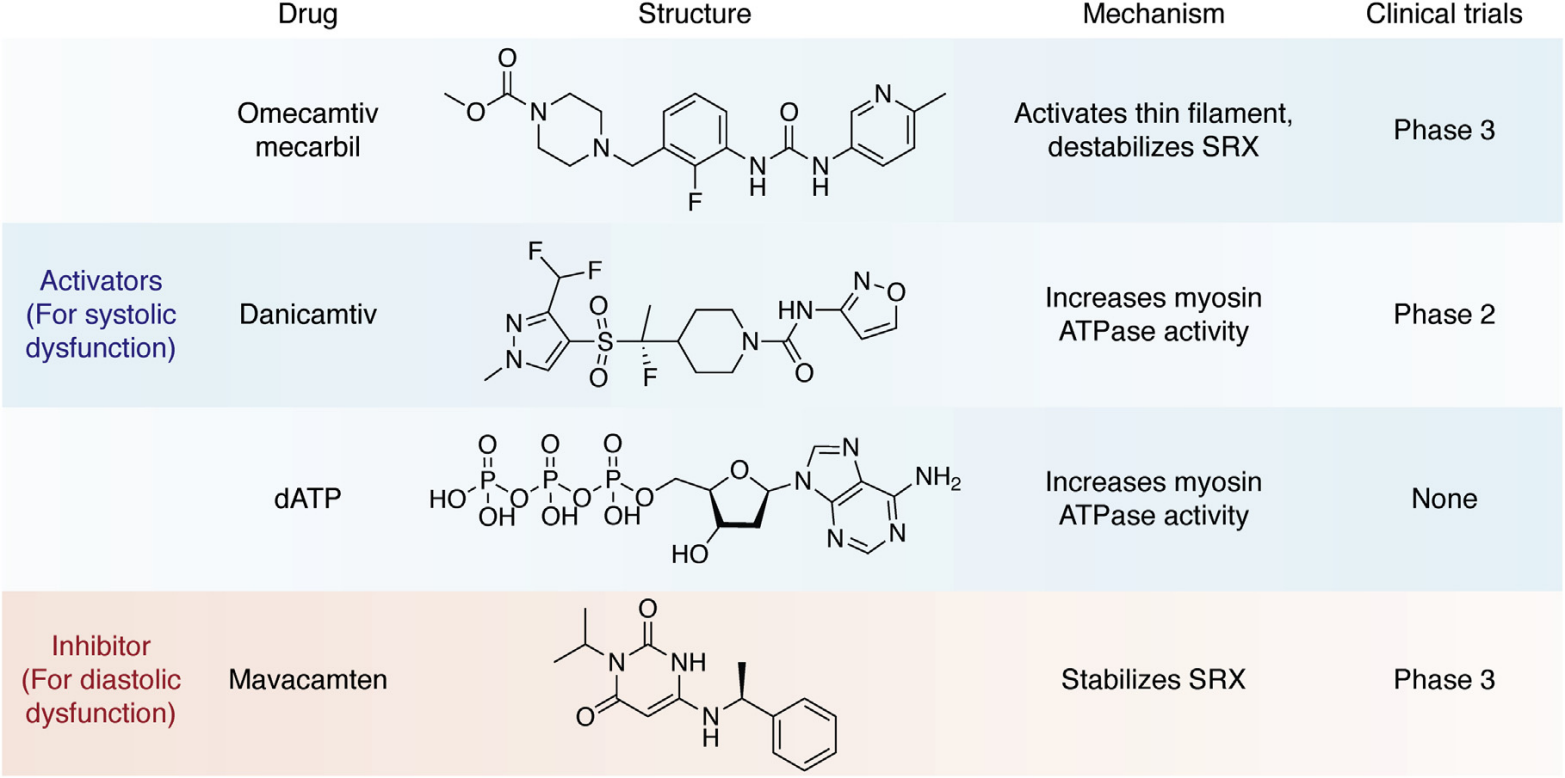
**Pharmacological agents targeting cardiac myosin.** Chemical structures, mechanisms, and current clinical trial status of myosin-targeting drugs that treat systolic (*blue shading*) or diastolic (*red shading*) dysfunction.

Due to its role in driving and regulating heart contraction, as well as its dysfunction in disease, cardiac myosin has emerged as a drug target. There are several myosin-binding compounds in development and in clinical trials that target cardiac contractility either directly or through its regulatory mechanisms (Fig. 4).

### Activators of cardiac contraction for systolic dysfunction

There are several conditions that can lead to heart failure with reduced systolic function, including coronary artery disease, myocardial infarction, myocarditis, and dilated cardiomyopathy (166). The majority of current therapeutics alleviate symptoms and target prevention of additional adverse remodeling; however, the transplant-free survival rate is low. Moreover, many of these therapeutics show limited efficacy in pediatric heart failure (167). Therefore, there is an outstanding clinical need to develop therapeutics to improve outcomes for patients, or patient subpopulations, with systolic heart failure.

Omecamtiv mecarbil (OM) was the first small-molecule therapy targeting cardiac myosin (168). It was discovered in a high-throughput screen for compounds that increase the actin-activated ATPase rate of cardiac myosin by increasing the rates of ATP hydrolysis and phosphate release. OM binds near the myosin converter domain, and therefore, exerts its effects on the nucleotide-binding pocket allosterically. Studies in isolated myocytes showed improved systolic function without changes to the calcium transient. Moreover, the recent GALACTIC-HF study demonstrated improved outcomes of patients with heart failure in phase 3 clinical trials (169).

Given its effects on the myosin ATPase rate, OM was originally proposed to act as a myosin activator; however, further studies revealed that its biophysical mechanism of action is more complicated than originally realized. While OM increases the actin-activated ATPase rate of unregulated thin filaments, it acts as an inhibitor when examined using regulated thin filaments at high calcium (170). Moreover, OM causes a dramatic ∼20-fold reduction in myosin’s motility rate, suggesting a complex mechanism of action (171). Recent optical trapping and transient kinetics experiments demonstrated that OM causes cardiac myosin to enter a noncanonical pathway with a long-lived strong binding state (25, 172). Moreover, OM-bound myosins do not generate a productive working stroke (25, 173). Thus, OM uncouples the kinetics and mechanics of the working stroke of individual motors, leading to reduced motile function.

Given that OM appears to reduce actomyosin detachment kinetics and inhibit the working stroke, how does it increase cardiac contractility in myocytes and patients? These seemingly contradictory results could be resolved by two nonexclusive mechanisms revealed by looking at systems of increasing complexity. The first mechanism involves thin filament activation. As mentioned earlier, thin filament activation can be accomplished by both calcium and myosin binding. By increasing the lifetime of the strongly bound state of cardiac myosin, OM keeps the thin filament in an activated state, leading to increased contractility (25). This result is supported by studies from muscle fibers showing that OM is most effective over a narrow range of dosages (174). At low OM concentrations, OM-bound crossbridges activate the thin filament while crossbridges without OM continue to power contraction, leading to increased force production. At higher concentrations of OM, the fraction of OM-bound unproductive crossbridges increases, reducing the force production. In this context, OM is better characterized as a thin filament activator rather than a myosin activator. In the second mechanism, OM decreases the fraction of myosin heads in the SRX, which increases force production by the muscle by increasing the number of available heads (175). These two mechanisms of OM action, thin filament activation and SRX destabilization, combine to increase cardiac contraction.

The effects of OM could be specific to mutations. An elegant example of this was the demonstration that despite suppressing the working stroke of wild-type myosin, OM rescues the reduced working stroke of myosin carrying the R712L mutation (21). This surprising finding was suggested to result from OM binding to the post-power stroke state of R712L myosin rather than the pre-power stroke state, highlighting the complexity of the therapeutic mechanism of OM and of myosin force generation more broadly. It also raises the possibility that OM may work for some mutations better than others, highlighting the importance of precision medicine approaches.

Recently, a new small-molecule compound, danicamtiv, was discovered as a myosin activator (176). While the details about this compound are still sparse, it appears to work by increasing the myosin ATP turnover rate, improving systolic performance without affecting diastolic tension (176, 177). Danicamtiv is currently in phase 2 clinical trials (177).

Another intriguing potential therapy for systolic heart failure is the use of deoxy-ATP (dATP) (178). In contrast to OM and danicamtiv, which were identified in high-throughput screens of small molecules that alter myosin contractility, dATP is a naturally occurring ATP analog. dATP binds to the myosin ATPase-binding site, where it can compete with the binding of ATP. The steady-state maximum actin-activated ATPase rate of myosin is higher for dATP than for ATP (178, 179). In contrast to OM, dATP increases the unloaded shortening velocity without affecting the isometric tension in muscle fibers (178). Although dATP binds to the myosin nucleotide-binding site, the effects of the drug on the structural dynamics of actomyosin are more subtle than initially thought. Molecular dynamics simulations revealed that binding of dADP and P_i_, the hydrolysis products of dATP, induces allosteric changes in the myosin actin-binding site that increase electrostatic interactions between myosin and actin, enhancing the crossbridge attachment rate (180). By increasing the attachment rate of myosin, this increases the number of bound, force-generating crossbridges.

One interesting aspect of dATP therapy is that dATP is a naturally occurring compound, produced by ribonucleotide reductase. As such, its therapeutic use is not confined to administering the compound directly to patients. Strategies to introduce dATP into cardiomyocytes *via* overexpression of ribonucleotide reductase have been described, demonstrating the potential of cardiac-specific delivery using gene therapy (181, 182, 183). Overexpression of dATP has been demonstrated to improve contractility in several animal models (184, 185, 186, 187), as well as in human patient-derived tissue (188).

### Inhibitors of cardiac contraction for HCM

As mentioned earlier, HCM is typically associated with hypercontractility, and therefore, compounds reducing contractility could help counteract the effects of disease-causing mutations. Most current treatments for HCM are palliative in nature. For patients with outflow tract obstruction, they are treated with myectomy and drugs, which improve hemodynamics (*e.g.*, beta blockers, calcium channel blockers, and disopyramide) while for nonobstructive patients, there are no therapies (189). Recently, a new small-molecule therapy, mavacamten (190), was identified from a high-throughput screen for compounds that decrease myosin’s ATPase rate in myofibrils. This compound directly and specifically binds to cardiac myosin. X-ray diffraction, biochemical, and electron microscopy studies have revealed that mavacamten decreases myosin contractility by stabilizing the SRX (68). As such, it decreases contractility by reducing the number of available myosin heads. Mavacamten has shown promising results in phase 3 clinical trials (191).

## Summary and future outlook

Although cardiac myosin has been thoroughly studied for decades, ongoing studies across scales of organization have revealed new mechanisms of myosin function and dysfunction that have enabled the development of promising therapeutic candidates. There are still many unanswered questions and challenges in this exciting field (Box 1). The structural and biochemical mechanisms by which myosin senses and transduces force are still largely unknown. Understanding of these mechanisms is crucial to understand the physiological role of cardiac myosin, as well as how dysregulation of myosin contraction and mechanotransduction contribute to cardiac disease. Furthermore, deeper understanding of disease mechanisms could guide the development of precision medicine approaches to treat patients. Although patient-specific treatments are likely unrealistic, it might be possible to bin mutations into groups that will positively respond to a given therapeutic (120). To realize the promise of this precision medicine approach, we also need to better understand the structural mechanisms of current and emerging drug compounds that target myosin function. Future work might also elucidate how specific mutations affect allosteric coupling within myosin, potentially expanding our ability to target cardiac myosin therapeutically. Addressing these gaps in our understanding will require the development of new experimental and computational methods that span scales of organization and faithfully recapitulate important aspects of myosin contractility. This is an exciting time for the field to ask basic science questions, with the possibility of translating these findings into improved treatment of devastating diseases.

Box 1 **Outstanding questions for the field**

**Table:** 

How is entry into the SRX regulated?
What structural mechanisms are involved in force sensing by myosin molecules and thick filaments?
Which signaling pathways are regulated by myosin mechanobiology, and how do they become dysregulated in disease?
How do posttranslational modifications of myosin and its binding partners tune myosin function over time during development and disease pathogenesis?
How do different disease-causing mutations in myosin lead to activation of different downstream pathways?
Can myosin mutations be binned into groups that will positively respond to a given therapeutic in order to improve patient outcomes through a precision medicine approach?
How do specific mutations affect allosteric coupling within myosin, and can this information explain why these myosin variants cause a given disease?
What are the structural mechanisms of the new drugs that target myosin function?

## Conflict of interest

The authors have no conflicts of interest to declare.
